# Contrasting the role of historic factors in phylogeograpic patterns in the native Johnny darter (*Etheostoma nigrum*) and invasive round goby (*Neogobius melanostomus*) in lower michigan

**DOI:** 10.1002/ece3.70232

**Published:** 2024-10-31

**Authors:** A. J. Wicks, M. Bowman, T. E. Dowling

**Affiliations:** ^1^ Department of Biological Sciences Wayne State University Detroit Michigan USA

**Keywords:** biogeography, freshwater fish, genetic distance, Great Lakes fishes, molecular dating

## Abstract

Round goby (*Neogobius melanostomus*) is an invasive fish present in all five Great Lakes and is becoming increasingly common in their tributaries. Johnny darter (*Etheostoma nigrum*) is a native species that often coexists with *N. melanostomus*. In this work, historic factors are addressed as a source of genomic variation in study populations of these species. To do this, patterns of variation in the mitochondrial gene NADH dehydrogenase subunit 2 (ND2) were characterized for both species throughout Lower Michigan. Populations of *N. melanostomus* and *E. nigrum* were sampled from 17 localities representing both eastern and western basins of Lower Michigan to test the hypothesis that populations differ between the eastern and western basins of the Great Lakes. *Neogobius melanostomus* populations were largely homogenous with no significant differences detected among populations or between the eastern and western basins. Additionally, *N. melanostomus* exhibited no evidence of overarching historical genetic structure, consistent with the recent invasion and rapid expansion of this species. *Etheostoma nigrum* exhibited significant differentiation among local populations; however, similarity among mtDNA haplotypes indicated that differences among populations are recent, suggesting that local forces are a more important factor in shaping patterns of variation than historical factors. Contrary to predictions, there were no significant differences detected between the eastern and western basins of the Great Lakes; however, construction of a neighbor‐joining tree with *F*_ST_ estimates revealed clustering of populations by basin with some anomalies. These anomalies may be the result of recent stream capture events facilitating gene flow between the two basins.

## Graphical abstract

Round goby (*Neogobius melanostomus*) is an invasive fish present in all five Great Lakes and is becoming increasingly common in their tributaries. Johnny darter (*Etheostoma nigrum*) is a native species that often coexists with *N. melanostomus*. In this work, historic factors are addressed as a source of genomic variation in study populations of these species using the mitochondrial gene NADH dehydrogenase subunit 2 (ND2) for populations throughout Lower Michigan.

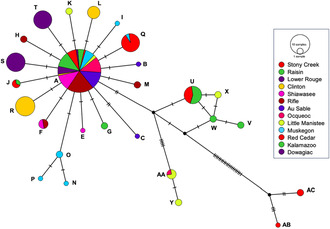

## 1 INTRODUCTION

Evolutionary history, geologic history, and ecology of a species contribute to variable patterns of genetic diversity across the landscape (e.g., Avise, 1992; Heithaus & Laushman, 1997; Leclerc et al., 2008). Quantifying these patterns provides information on the structure and dynamics of populations in the context of landscape features and how historical factors can influence variation in the genome and gene expression. Depending on an organism's dispersal ability, landscape features may function to facilitate gene flow or create barriers that isolate populations. Isolated populations become genetically distinct over time, proceeding on independent evolutionary tracks due to mutation, drift, and differential selection. However, if connections exist that facilitate dispersal, populations can experience continuous gene flow, potentially eliminating differences among them (Slatkin, 1987). Species vary in their ability to disperse and the extent to which landscape features facilitate or prevent gene flow depends on the ecology of a species (Hitt & Angermeier, 2008). Taxa with more limited habitat preferences may be restricted to suitable areas (Tibbets & Dowling, 1996) while a more plastic species may be able to disperse through variable habitats; therefore, patterns of genetic diversity are likely to reflect these ecological differences (Bohonak, 1999).

The recent glacial history of the Great Lakes region has influenced population structure and the distribution of genetic diversity in its native fishes. The Laurentian Great Lakes were formed by glacial action during the Pleistocene, approximately 14,000 years ago, created by retreat of the Laurentide ice sheet (reviewed in Bailey & Smith, 1981). The Lake Michigan and Lake Erie/Huron basins were formed due to the retreat of separate glacial lobes, each with their own periglacial lakes (Lakes Chicago and Maumee, respectively) and outflow systems. Thus, the eastern and western basins of the Great Lakes were potentially colonized by fishes from distinct source populations that derived from separate glacial refugia (Bailey & Smith, 1981; Lewis et al., 2008). Population structure of fishes in rivers of the two basins often reflects this geologic history as some species from these basins are distinct relative to each other (e.g., Dowling et al., 1997; Gach, 1996; Stepien et al., 2009). Ecological factors influence observed patterns as well. Coldwater fishes may maintain populations near the glacial front where more recent connections could result in a more homogenous population colonizing the eastern and western basins of the post‐glacial Great Lakes (Bailey & Smith, 1981).

Here, population and phylogenetic approaches were used to characterize levels of genetic diversity and population structure in Lower Michigan for two common Great Lakes fishes, round goby (*Neogobius melanostomus*) and Johnny darter (*E. nigrum*), to better understand factors that contribute to patterns of variation in these two species. Indirect and direct evidence of negative interactions between these species has been reported. *Etheostoma nigrum* has been shown to decline in abundance as *N. melanostomus* becomes established (Lauer et al., 2004; Burkette & Jude, 2015), and in experimental enclosures, *E. nigrum* have exhibited decreased growth in coexistence with round goby (Kornis et al., 2014). This negative interaction is not absolute, however, as Johnny darter and round goby have been found to coexist without evidence of negative impacts (Riley et al., 2008; Kornis, Sharma, & Vander Zanden, 2012).

The variable impacts of *N. melanostomus* on *E. nigrum* populations have made the two species an interesting case study for understanding the factors that contribute to success of invasive species and their impacts on natives. Next generation sequencing technology has made it possible to study ecological interactions through quantification of patterns of gene expression (Ekblom & Galindo, 2010), providing a perspective of how physiological processes can change in varying environments of natural systems. Comparing gene expression patterns could be informative in understanding the distribution of *N. melanostomus* in the Great Lakes region and impacts on interactions with native species like *E. nigrum*; however, knowledge of evolutionary history is important because differences in their evolutionary history may have a significant influence on differences in local patterns of gene expression. The present study serves as a necessary precursor to future work characterizing patterns of gene expression in these species. Although many studies have characterized population structure of *N. melanostomus* in the Great Lakes (e.g. Brown & Stepien, 2008; LaRue et al., 2011; Snyder & Stepien, 2017), population genetic studies of the *N. melanostomus* populations colonizing inland lakes and streams are fewer, particularly in Lower Michigan; there are no previous population genetic studies of *E. nigrum* in Lower Michigan. Given the recent ages of these populations, local evolutionary forces are more likely to play a significant role in gene expression patterns for round goby and Johnny darter than would the influence of genetic differences accumulated in older populations.

As a native species to the Great Lakes, *E. nigrum* populations may exhibit patterns that reflect their colonization history as glaciers retreated. As such, populations from the western basin of the Great Lakes may be significantly different from those of the eastern basin, illustrating how historical processes can influence current patterns of genetic variation. Natural and anthropogenic barriers are also likely to influence structure in *E. nigrum* populations as *E. nigrum* prefer shallow, slow‐moving water; therefore, large waterways may function as barriers to gene flow and could result in differentiation across study rivers (Leidy, 1992).

Unlike *E. nigrum*, *N. melanostomus* is a recent invader of the Great Lakes. It was first discovered in the St. Clair River in 1990 (Jude et al., 1992), therefore, this species is not influenced by local geological history. Population genetic studies using microsatellite and mitochondrial DNA markers determined that the Great Lakes were populated by individuals derived from a single source population from a tributary to the Black Sea (Brown & Stepien, 2009), and *N. melanostomus* is now present in all five Great Lakes and in many tributaries (Kornis, Mercado‐Silva, & VanderZanden, 2012). Analyses of population structure indicate that genetic diversity of the source population is largely maintained throughout the Great Lakes; some limited structure has been identified and is primarily driven by differences in the populations of Saginaw Bay and Lake Ontario (Brown & Stepien, 2009). In recently colonized Great Lakes tributaries, *N. melanostomus* populations have generally come to represent their local source populations likely due to consistent propagule pressure through migration and aided by human activity (Bronnenhuber et al., 2011; Sard et al., 2019).

As a recent invader, *N. melanostomus* are expected to exhibit limited differences (e.g., numbers of haplotypes and mutations among them) within and among sampled populations. Additionally, it is expected that recently established riverine populations may exhibit only a subset of the variation found in the source lake populations (Brown & Stepien, 2008). *Etheostoma nigrum* has resided in this region since glaciation (Bailey & Smith, 1981), and given the complex glacial history of the region, there may be differences within and/or among regions (e.g., eastern vs. western Great Lakes). As a native species, *E. nigrum* populations are more likely to have diverged since their colonization as compared to *N. melanostomus*, thus more genetic differentiation is expected among populations of *E. nigrum* than *N. melanostomus*. Because of their propensity to inhabit shallow streams, large waterways are also likely to represent a barrier to gene flow for *E. nigrum* and could lead to divergence among populations. These hypotheses were tested by sequencing the mitochondrial DNA gene NADH dehydrogenase subunit 2 (ND2) for populations of *E. nigrum* and *N. melanostomus* throughout lower Michigan.

## 2 METHODS

**FIGURE 1:**
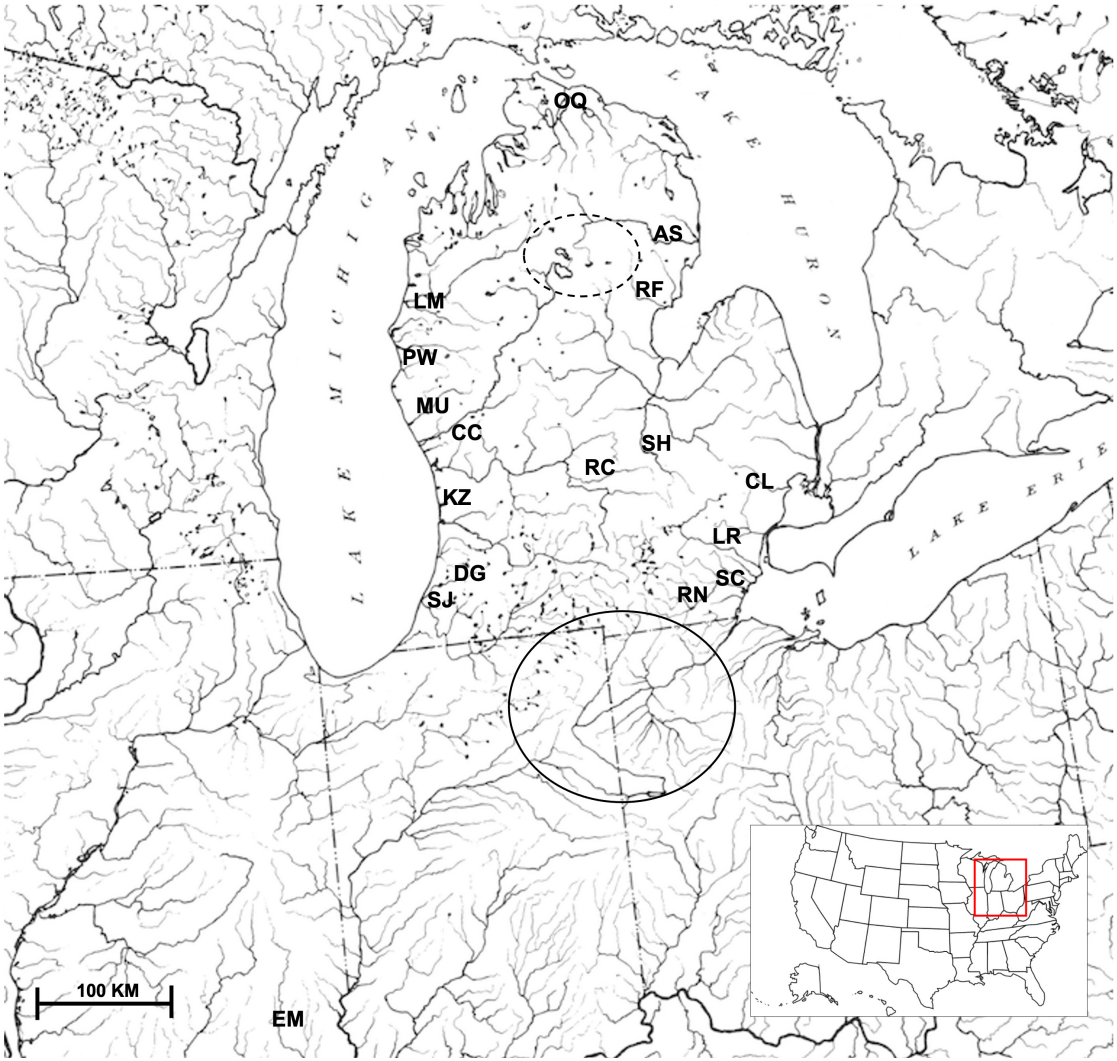
Map of collection localities for *N. melanostomus* and *E. nigrum*. Acronyms for sampling localities are: Au Sable (AS), Clinton (CL), Crockery Creek (CC), Dowagiac (DG), Kalamazoo (KA), Little Manistee (LM), Lower Rouge (LR), Muskegon (MU), Oqueoc (OQ), Pentwater (PW), Raisin (RN), Red Cedar (RC), Rifle (RF), St. Joseph (SJ), Shiawasee (SH), Stony Creek (SC). Precise location information is provided in the Methods section. A sequence of *E. nigrum* from the Embarras River (EM) in Illinois was obtained from GenBank. The dotted circle is the location of the headwaters of the Muskegon River and the Au Sable River. The solid circle shows the region where the Maumee River captured a portion of the Wabash River. Base map is reprinted from the Fish Division drainage map, University of Michigan Museum of Zoology, under a CC BY license, with permission from University of Michigan Museum of Zoology, original copyright 1972.

Sixteen stream localities were selected representing the Lake Michigan, Lake Huron, and Lake Erie watersheds (Figure 1). Round goby was first identified in Lake Erie and Lake Michigan in 1993 and Lake Huron in 1997 (Clapp et al., 2001; Schaeffer et al., 2005). Fishes were collected by seining, with up to 20 individuals of each species collected at each site. Acronyms used in tables and figures for sampling localities are: Au Sable (AS, 44.503294, −83.793609), Clinton (CL, 42.671619, −83.095602), Crockery Creek (CC, 43.053465, −86.062942), Dowagiac (DG, 42.011859, −85.962827), Kalamazoo (KA, 42.638600, −86.163315), Little Manistee (LM, 44.209024, −86.263904), Lower Rouge (LR, 42.285374, −83.388700), Muskegon (MU, 43.297940, −86.079321), Oqueoc (OQ, 45.456219, −84.087664), Pentwater (PW, 43.769223, −86.424267), Raisin (RN, 41.922478, −83.695259), Red Cedar (RC, 42.698207, −84.404845), Rifle (RF, 44.141451, −84.043657), St. Joseph (SJ, 42.074666, −86.461342), Shiawasee (SH, 42.919861, −83.969519), Stony Creek (SC, 42.023489, −83.419425). In some localities where one or both species was rare, multiple nearby localities and/or sample dates were combined to achieve desired sample size. As both species were not present at every locality, the Dowagiac, Raisin, and Shiawasee include only *E. nigrum*. Crockery Creek, Pentwater, and St. Joseph include only *N. melanostomus*. Individuals were fin clipped, and tissue was stored in 95% ethanol. For DNA isolation, tissue samples were dissolved in a solution of proteinase K and sodium dodecyl sulfate and purified via one of two methods: phenol/chloroform extraction method or magnetic bead DNA purification (samples collected in 2015–2016 and 2017–2019, respectively). Phenol/chloroform extraction followed Tibbets and Dowling (1996). Magnetic bead purifications followed the manufacturer's (Axygen) protocol with the following changes: 25 μL of lysate was added to 25 μL of AxyPrep, 70% ethanol was used for two washes, and DNA was eluted in 40 μL of water. DNA was assessed for quality and quantity with a Nanodrop Spectrophotometer (ThermoFisher).

The mitochondrial gene NADH dehydrogenase subunit 2 (ND2) was selected because of its relatively high rate of mutation and strict maternal inheritance, allowing for better characterization of more recent events (Avise et al., 1987; Mueller, 2006). Sequences were amplified using the primers B2Gila (5′‐CTCTTAGTGCTTCCTCACA‐3′) and ASN (5′‐CGCGTTTAGCTGTTAACTAA‐3′) for *E. nigrum* and RGND2F (5′‐AGCATGCCGGTTAAAATCC‐3′) and RGND2R (5′‐GGATCCGAGGCCTTCCTGTCT‐3′) for *N. melanostomus*. The following PCR conditions were used for both species: 94°C for 15 min, 25 cycles of denaturation at 94°C for 1 min, annealing at 58°C for 1 min, and polymerization at 72°C for 2 min with a final annealing step of 72°C for 10 min, and holding at 4°C until long term storage at −20°C. Amplification products were checked for quantity and quality using agarose gel electrophoresis prior to being sent to the Wayne State University's Applied Genomics Technology Center or Eton Bioscience for 2015–2018 and 2019 samples, respectively. Products were sequenced with the same primers using Applied Biosystems DNA Analyzer 3730 sequencer, yielding total of 1047 bp (the entire ND2 gene) for *E. nigrum*. Due to low‐quality reads at the beginning of the gene for some samples, *N. melanostomus* sequences were trimmed to 1029 bp.

Some individuals included in this study were also included in a separate RNA‐seq study (Wicks, 2020). For these individuals, ND2 gene sequences were obtained from RNA‐seq reads. From the assembly and aligned read files, the SAMtools package (Li, 2011) was used to call SNPs and output as a consensus sequence for each individual. Variants were quality filtered for phred score >30 and individuals with ambiguous variant calls were excluded from the analysis.

Sequences were aligned, assembled, and trimmed in Bioedit (version 7.0.5.3) (Hall et al., 2011). MEGA (version 5.2.2) (Kumar et al., 2018) was used to produce a maximum‐likelihood tree and to assign haplotypes. Sequences and haplotype counts for each location were used in ARLEQUIN (version 3.5.1.2) (Excoffier & Lischer, 2010) to obtain estimates of genetic diversity (e.g., number of haplotypes, gene diversity) within populations. Variation among populations was assessed and tested for significance at *α* = .05 using a molecular analysis of variance (AMOVA), also in ARLEQUIN. This approach was used to partition levels of genetic variation within and among river drainages and regions (e.g., eastern vs. western basins of the Great Lakes). Haplotype networks were created as median‐joining networks with PopArt (version 1.7) (Leigh & Bryant, 2015). Neighbor‐joining trees of populations were generated with pairwise *F*_ST_ values in MEGA. For *E. nigrum*, additional ND2 sequences were obtained from NCBI GenBank and included in the maximum‐likelihood tree of haplotypes, also generated with MEGA with 1000 bootstrap replicates. These included closely related species *E. olmstedi* (EF027210), *E. podostemone* (JQ088571), *E. perlongum* (JQ088568), *E. susanae* (JQ088589), *E. vitreum* (FJ381264), and *E. longimanum* (JQ088552) and co‐occurring darter species *E. blennioides* (JQ088546), *E. exile* (EF027194), and *E. caereleum* (JQ088546). Additionally, one *E. nigrum* ND2 sequence (JQ088561) from an individual collected from the Embarras River, a tributary of the Wabash River (N. Lang [personal communication, 11 November 2019]) was obtained from GenBank and included in the analysis.

To provide context for the large divergence among some *E. nigrum* haplotypes, the program *BEAST (found within BEAST 2 v2.7.3, Bouckaert et al., 2014) was used to estimate divergence dates among *E. nigrum* haplotypes as well as other *Etheostoma* species included in the maximum‐likelihood tree. *Etheostoma* species are not well represented in the fossil record (Bailey & Smith, 1981); therefore, previous estimates of molecular divergence in darters that have used Centrarchid fossil records for calibration for divergence dating were used here (e.g., Bossu et al., 2013; Fluker et al., 2014; Near et al., 2011). The substitution rate was estimated by Near et al. (2011) from the mitochondrial cytochrome b (cyt*b*) gene as 8.99 × 10^−3^, and this has been applied in recent estimates of molecular divergence in *Etheostoma* species (Echelle et al., 2015; MacGuigan et al., 2023; McCall & Fluker, 2021). Although this rate may result in an underestimate of divergence time for ND2 as ND2 evolves at a faster rate than cyt*b* (Mueller, 2006), it is the best available rate for estimating divergence time from mitochondrial genes in *Etheostoma* species and was therefore used as the substitution rate in this analysis. *BEAST was run using default options except for the use of a species tree relaxed clock, HKY substitution model, and Yule model as a tree prior. Within the BEAST package, TreeAnnotator was used to generate a maximum clade credibility tree using median node heights and the tree was visualized with FigTree.

## 3 RESULTS

### 3.1 Neogobius melanostomus

**FIGURE 2:**
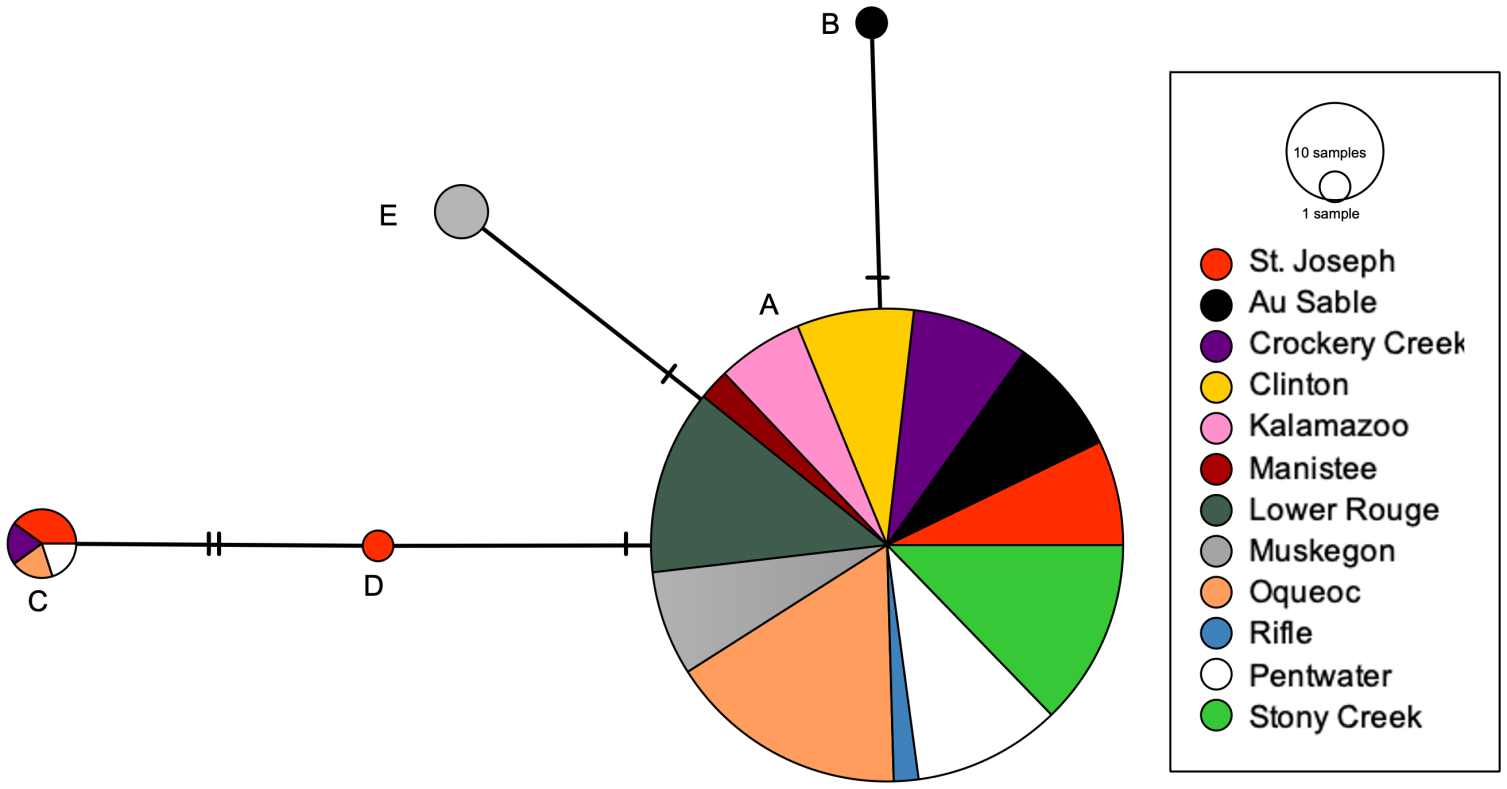
Median‐joining network of *N. melanostomus* haplotypes. Pies show proportional representation of haplotypes by locality (indicated by different colors), with size of the circle reflecting the number of individuals. Mutations between haplotypes are indicated by vertical lines.

A total of 247 *N. melanostomus* samples were collected from 12 localities and sequenced for ND2. Five haplotypes were identified (Table 1; Figure 2). Each was separated by one substitution from its closest neighbor except for haplotype C, which differed by two changes. Four variants resulted from changes to third codon positions (Haplotypes A–D) and did not result in amino acid changes. One variant resulted from a mutation in a first codon position and resulted in an amino acid change (Haplotype E).

**TABLE 1:** Sample size (*N*) and haplotype counts (identified as letters) for samples of *N. melanostomus*

Major drainage	Locality	*N*	Haplotype				
			A	B	C	D	E
Lake Erie	Stony Creek	30	30				
	Lower Rouge	30	30				
Lake St. Clair	Clinton	19	19				
Lake Huron	Rifle	4	4				
	Au Sable	20	19	1			
	Oqueoc	40	39		1		
Lake Michigan	Manistee Lake	5	5				
	Pentwater Lake	25	24		1		
	Muskegon	20	17				3
	Crockery Creek	20	19		1		
	Kalamazoo	14	14				
	St Joseph	20	17		2	1	
Total		247	237	1	5	1	3

*Note*: Complete locality data are provided in Section 2.

**FIGURE 3:**
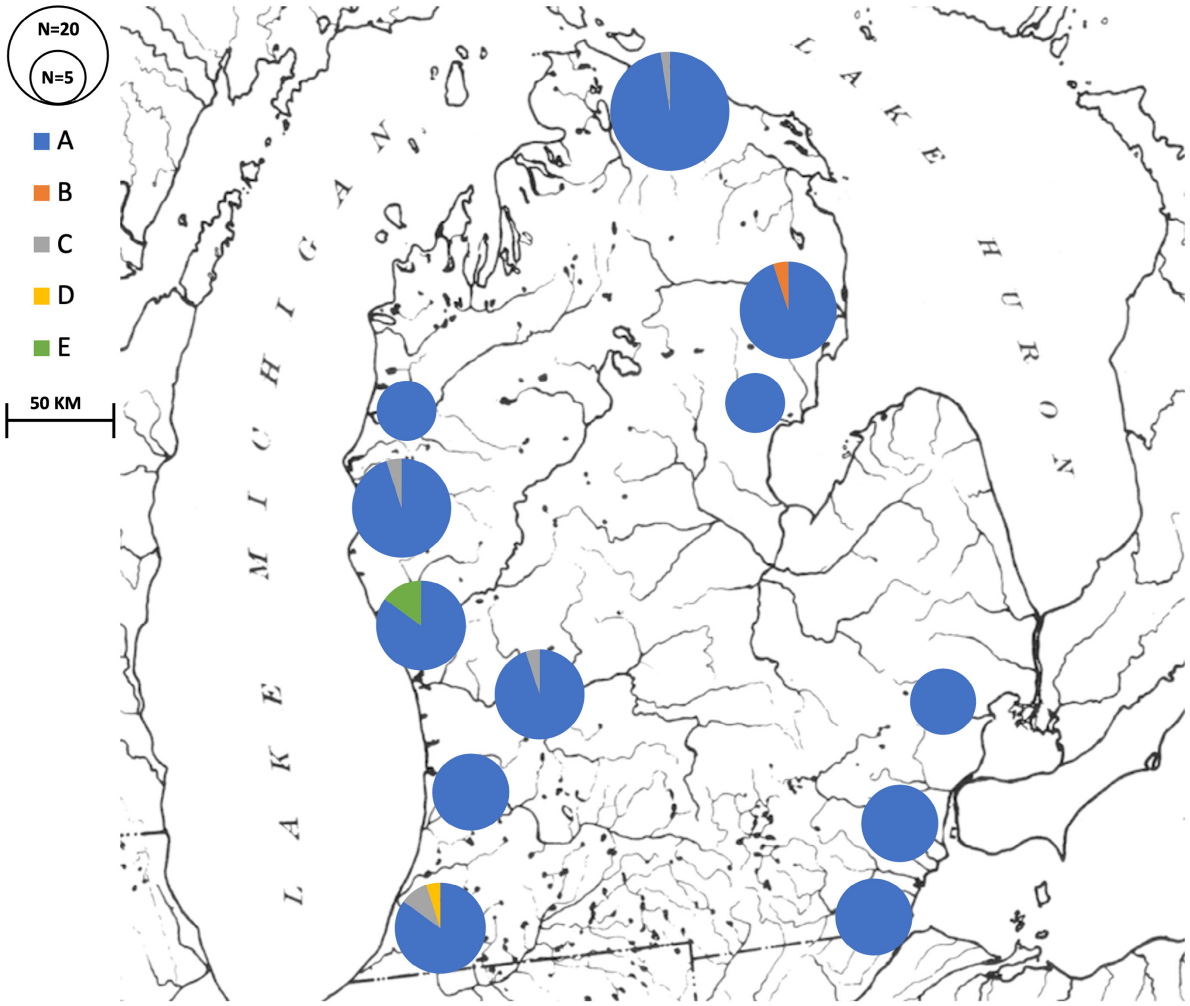
Distribution of haplotypes among sampling localities for *N. melanostomus*. The size of the circle reflects sample sizes, and the haplotype frequency is reflected by size of different colored slices. Haplotype colors are identified in the legend at the upper left.

**FIGURE 4:**
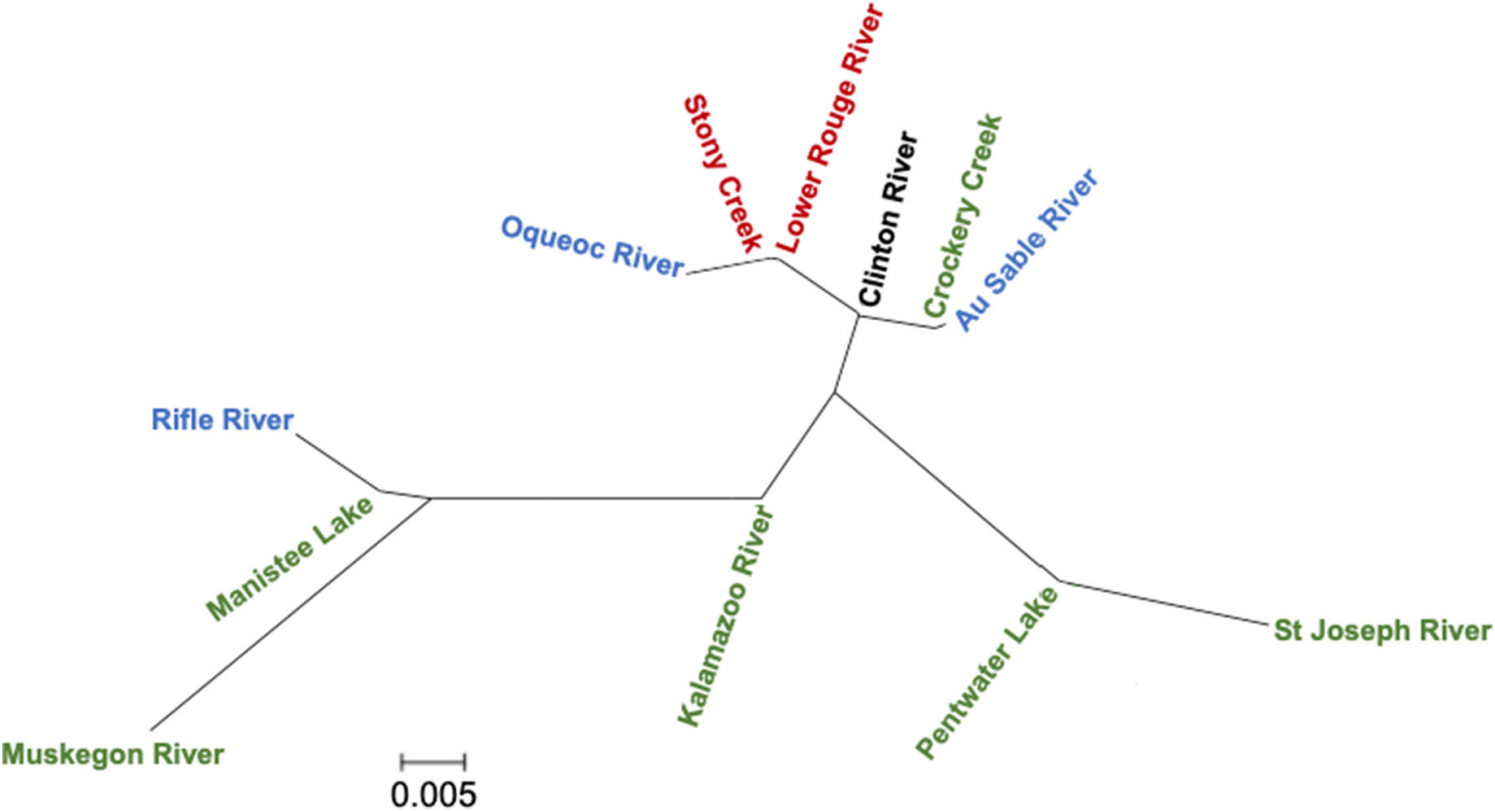
Neighbor‐joining tree based of pairwise *F*_ST_ estimates for *N. melanostomus*. Color indicates major drainage: Black, Lake St. Clair; Green – Lake Michigan; Red – Lake Erie; Blue – Lake Huron.

Haplotype A was found in 96% of all individuals sampled, resulting in very low levels of gene and nucleotide diversity within samples (Table 2; Figure 3). AMOVA was used to quantify the levels of genetic variance within and among samples (*F*_ST_). The distribution of variation between the lower Great Lakes was assessed by further partitioning variance between tributaries flowing into eastern (Lakes Huron, St. Clair, and Erie) and western lakes (Lake Michigan) (*F*_CT_) and among samples within these two groups of drainages (*F*_SC_). Statistical assessment failed to identify significant differences among samples (*F*_ST_ = 0.033, *p* = .106), between the two drainages (*F*_CT_ = 0.017, *p* = .055), or among samples within these two drainages (*F*_SC_ = 0.016, *p* = .259). This lack of differentiation was also supported by the examination of pairwise estimates of *F*_ST_ as only three of the comparisons were significant (Table 3). Similarity among samples was examined by clustering samples by *F*_ST_ using the neighbor‐joining method (Figure 4). There was no overarching genotypic structure of *N. melanostomus* populations as samples from different lake basins were intermingled.

**TABLE 2:** Estimates of molecular diversity in samples of *N. melanostomus* for each of the drainages sampled.

Drainage	*N*	*N*_h_	Gene diversity	Mean No. of pairwise differences	Nucleotide diversity
Stony Creek	30	1	0.0000 ± 0.0000	0.000000 ± 0.000000	0.000000 ± 0.000000
Lower Rouge	30	1	0.0000 ± 0.0000	0.000000 ± 0.000000	0.000000 ± 0.000000
Clinton	19	1	0.0000 ± 0.0000	0.000000 ± 0.000000	0.000000 ± 0.000000
Rifle	4	1	0.0000 ± 0.0000	0.000000 ± 0.000000	0.000000 ± 0.000000
Au Sable	20	2	0.1000 ± 0.0880	0.100000 ± 0.177536	0.000097 ± 0.000193
Oqueoc	40	2	0.0500 ± 0.0469	0.150000 ± 0.216477	0.000146 ± 0.000234
Manistee Lake	5	1	0.0000 ± 0.0000	0.000000 ± 0.000000	0.000000 ± 0.000000
Pentwater Lake	25	2	0.0800 ± 0.0722	0.240000 ± 0.284615	0.000233 ± 0.000308
Muskegon	20	2	0.2684 ± 0.1133	0.268421 ± 0.305890	0.000261 ± 0.000332
Crockery Creek	20	2	0.1000 ± 0.0880	0.300000 ± 0.326275	0.000292 ± 0.000354
Kalmazoo	14	1	0.0000 ± 0.0000	0.000000 ± 0.000000	0.000000 ± 0.000000
St. Joseph	20	3	0.2789 ± 0.1235	0.647368 ± 0.523752	0.000629 ± 0.000568

*Note*: *N* and N_h_ refer to the number of individuals and number of haplotypes per sample, respectively. Standard deviations are provided for each estimate.

**TABLE 3:** Pairwise estimates of *F*_ST_ for populations of *N. melanostomus*.

	SC	CC	KZ	LM	MU	PW	AS	CL	LR	OQ	RF
CC	−0.0187										
KZ	0.04749	−0.019									
LM	−0.0477	−0.1047	0								
MU	0.08421	0.05263	0.07447	−0.0241							
PW	0.00095	−0.0459	−0.0255	−0.107	0.05935						
AS	0.06579	0	−0.019	−0.1047	0.07895	−0.004					
CL	0.07109	−0.0026	0	0	0.10065	−0.0115	−0.0026				
LR	0.11047	0.02104	0	0	0.14537	0.00744	0.02104	0			
OQ	−0.0462	−0.0289	−0.0326	−0.1095	0.08392	−0.0297	−0.0048	−0.021	−0.0074		
RF	0.07989	−0.1377	0	0	−0.0559	−0.1377	−0.1377	0	0	−0.1416	
SC	0.11047	0.02104	0	0	0.14537	0.00744	0.02104	0	0	−0.0074	0

*Note*: Significant values are underlined. See Section 2 for locality abbreviations.

### 3.2 Etheostoma nigrum

**FIGURE 5:**
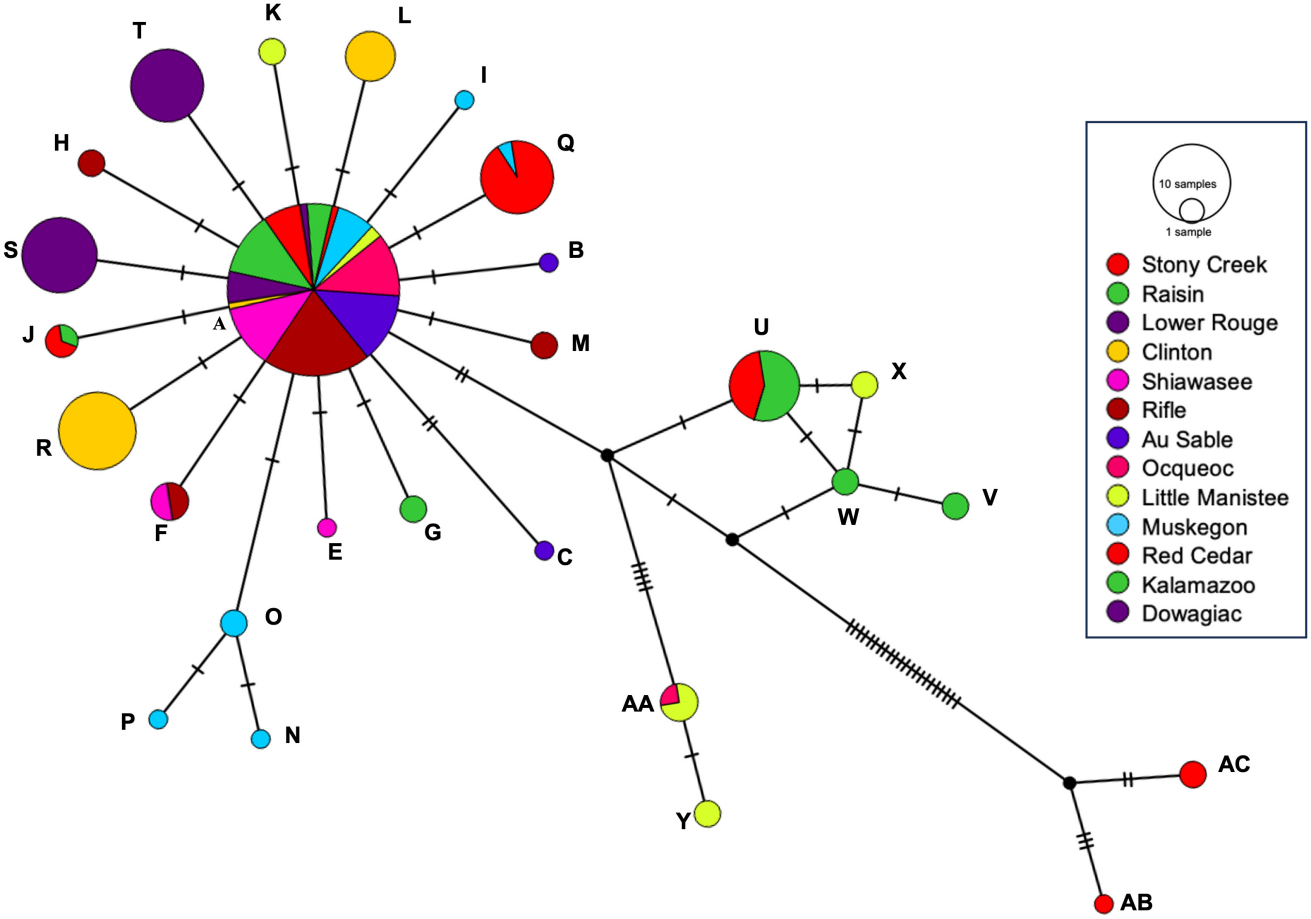
Median‐joining network of *E. nigrum* haplotypes. Pies show proportional representation of haplotypes by locality, and size of the circle reflects number of individuals with that haplotype. Mutations between haplotypes are indicated by vertical lines, and the frequency of each haplotype is reflected by size of different colored slices, which are identified in the legend at the right.

A total of 207 *E. nigrum* individuals were collected from 13 localities and sequenced for ND2. Twenty‐eight haplotypes were identified (Table 4; Figure 5). There were 53 polymorphic sites with 13 of those variants in the first codon position and 40 in the third codon position. Of these, 10 changes resulted in amino acid changes. Most of variants resulted from single base pair changes from the most common haplotype, A. Two haplotypes, AA and AB, differed from haplotype A by 28 and 27 base pair changes, respectively.

**TABLE 4:** Sample size (*N*) and haplotype counts (identified as letters) for samples of *E. nigrum*.

Major basin	Locality	*N*	Haplotype																											
			A	B	C	D	E	F	G	H	I	J	K	L	M	N	O	P	Q	R	S	T	U	V	W	X	Y	AA	AB	AC
Lake Erie	Stony Creek	17	6									2											6						1	2
	Raisin	13	10						2			1																		
	Lower Rouge	21	5																			16								
Lake St. Clair	Clinton	25	1											7						17										
Lake Huron	Shiawasee	14	10				2	2																						
	Rifle	23	17					2		2					2															
	Au Sable	13	11	1	1																									
	Oqueoc	11	10																									1		
Lake Michigan	Little Manistee	11	2										2													2	2	3		
	Muskegon	12	6								1					1	2	1	1											
	Red Cedar	15	1																14											
	Kalamazoo	16				4																	8	2	2					
	Dowagiac	16	1																		15									
Total		207	80	1	1	4	2	4	2	2	1	3	2	7	2	1	2	1	15	17	15	16	14	2	2	2	2	4	1	2

*Note*: Complete locality data are provided in the Section 2.

**FIGURE 6:**
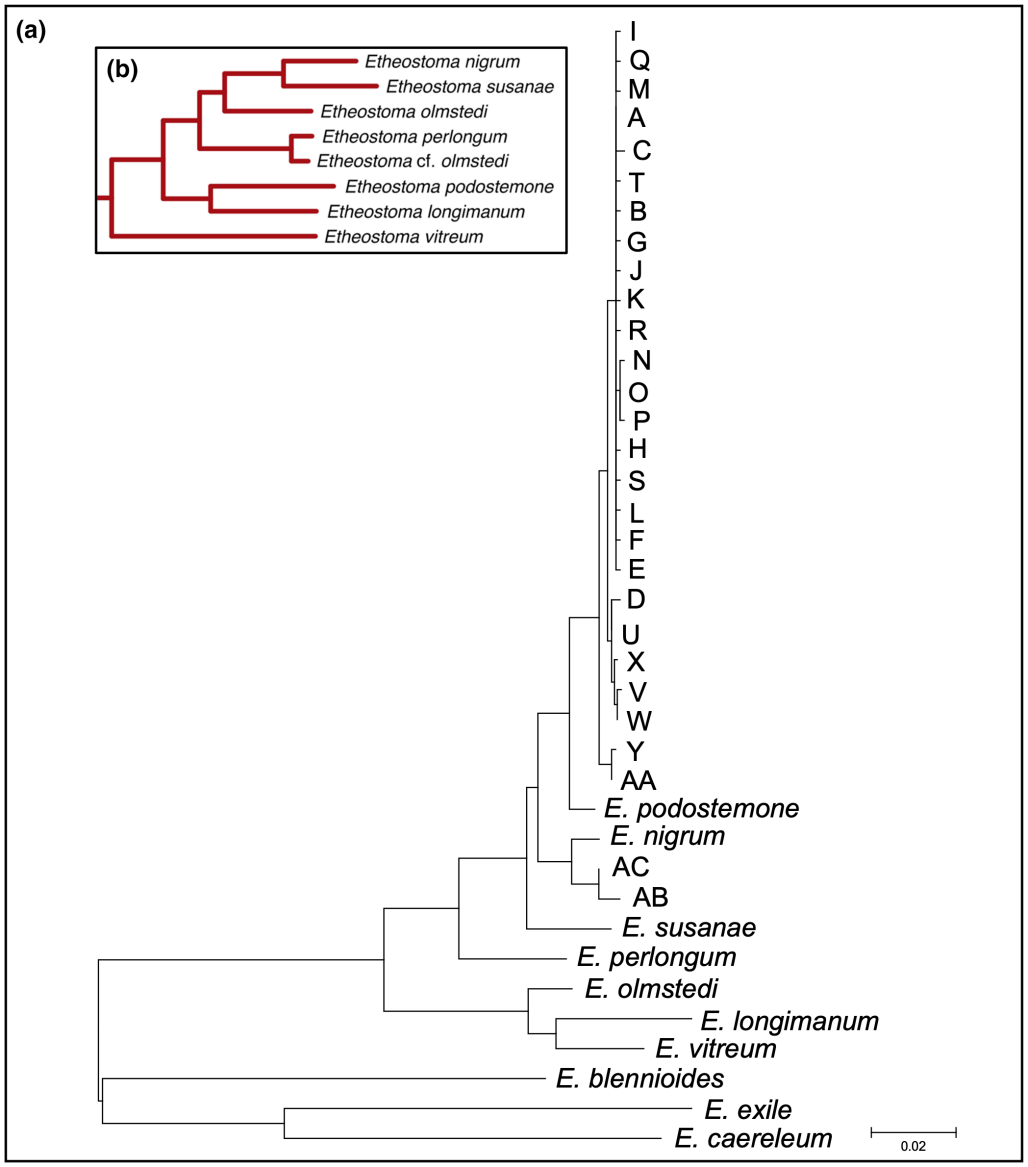
(a) Maximum‐likelihood tree of *E. nigrum* haplotypes with bootstrap values indicated. The tree includes closely related species *E. olmstedi, E. podostemone*, and *E. perlongum*, *E. susanae, E. vitreum*, *E. longimanum* and more distantly related but co‐occurring darter species *E. blennioides, E. exile*, and *E. caereleum*. The additional *E. nigrum* individual obtained from GenBank was sampled from the Wabash River drainage. (b) Phylogeny based on single nucleotide polymorphisms modified from MacGuigan and Near (2018).

A maximum‐likelihood tree was generated to examine haplotypic variation in *E. nigrum* from the lower peninsula of Michigan in phylogenetic context relative to other samples obtained from GenBank (Figure 6). Most haplotypes sampled cluster closely to the most common haplotype, A, and formed a well‐supported monophyletic lineage (98% bootstrap value) with limited phylogenetic structure among haplotypes within this group. Haplotypes D, U, V, W, and X form a lineage in the minimum spanning network (Figure 5) and form a monophyletic group in the maximum‐likelihood tree (Figure 6). This group exhibited a moderately high bootstrap value in the ML analysis (74%) and is found in samples from three separate drainages from both eastern and western Great Lakes (Stony Creek [Erie], Ocqueoc [Huron] and Kalamazoo [Michigan] rivers, Table 4). Another distinct haplotype lineage Y‐AA occurred only in the Oqueoc and Little Manistee rivers (Lakes Huron and Michigan drainages, respectively), and this lineage clusters closely to the unresolved large group of *E. nigrum* haplotypes. *Etheostoma podostemone* was the sister taxon to most haplotypes from the Great Lakes sampled here (98% bootstrap value). The two divergent haplotypes AB and AC from Stony Creek (Lake Erie drainage) share their most recent ancestor with *E. nigrum* from the Embarras River (Wabash River drainage) in central Illinois (95% bootstrap value).

**FIGURE 7:**
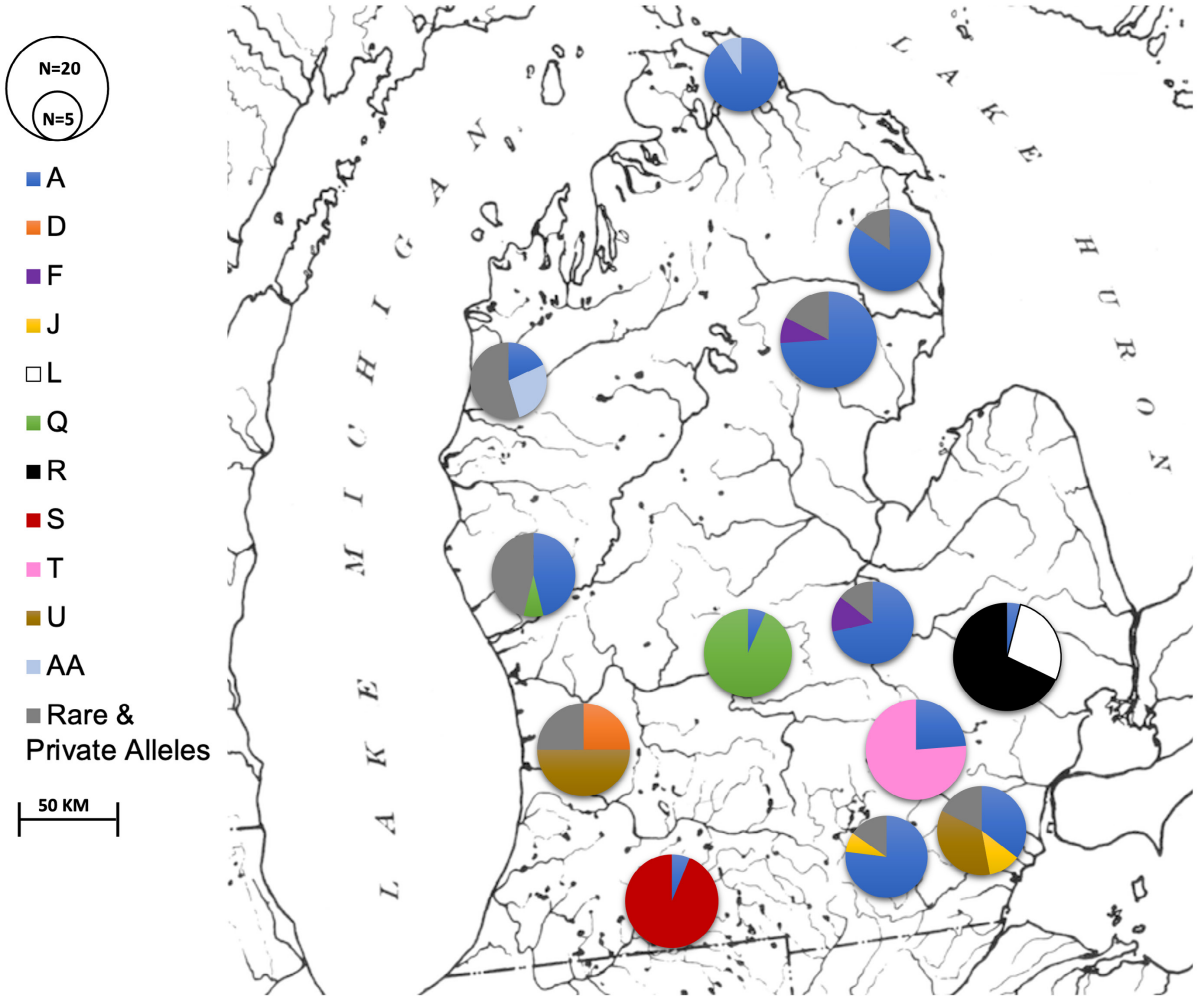
Distribution of haplotypes among sampling localities for *E. nigrum*. The size of the circle reflects sample size. The frequency of each haplotype is reflected by size of different colored slices, which are identified in the legend at the left. For ease of presentation, alleles that are both rare and private were collapsed into one category. Precise allele counts are available in Table 4.

The levels of variation within and among samples were used to assess geographic population structure in *E. nigrum* (Figure 7). Haplotype A was the most common, found in more than 40% of all individuals sampled and at every locality except the Kalamazoo River. All other haplotypes were more localized, occurring at a maximum of two localities. Private alleles were also common, with 22 alleles occurring at only single localities; however, every locality harbored more than one haplotype.

The levels of sequence diversity were highly variable among samples (Table 5), with gene diversity and number of pairwise differences ranging from 0.13 to 0.76 and 0.13 to 9.66, respectively. Exceptional levels of gene and nucleotide diversity were noted in Stony Creek and Little Manistee samples due to the presence of divergent haplotypes discussed above. Stony Creek samples exhibited five haplotypes with a mean number of pairwise differences of 9.67 relative to the other Great Lakes samples, and the Little Manistee River contained five haplotypes with a mean number of pairwise differences of 5.05.

**TABLE 5:** Estimates of molecular diversity for each sample of *E. nigrum*.

Drainage	*N*	*N*_h_	Gene diversity	Mean No. of pairwise differences	Nucleotide diversity
Stoney Creek	17	5	0.7647 ± 0.0657	9.661765 ± 4.659285	0.009219 ± 0.004976
Raisin	13	3	0.4103 ± 0.1539	0.435897 ± 0.416881	0.000416 ± 0.000447
Lower Rouge	21	2	0.3810 ± 0.1005	0.380952 ± 0.375176	0.000364 ± 0.000400
Clinton	25	3	0.4767 ± 0.0855	0.873333 ± 0.634421	0.000833 ± 0.000675
Shiawasee	14	3	0.4835 ± 0.1425	0.527473 ± 0.467361	0.000503 ± 0.000501
Rifle	23	4	0.4506 ± 0.1208	0.498024 ± 0.440699	0.000475 ± 0.000469
Au Sable	13	3	0.2949 ± 0.1558	0.461538 ± 0.431834	0.000440 ± 0.000463
Oqueoc	11	2	0.1818 ± 0.1436	1.272727 ± 0.864354	0.001214 ± 0.000930
Little Manistee	11	5	0.8727 ± 0.0593	5.054545 ± 2.657675	0.004823 ± 0.002861
Muskegon	12	6	0.7576 ± 0.1221	1.151515 ± 0.798643	0.001099 ± 0.000858
Red Cedar	15	2	0.1333 ± 0.1123	0.133333 ± 0.209858	0.000127 ± 0.000225
Kalamazoo	16	4	0.7000 ± 0.0896	1.433333 ± 0.921163	0.001368 ± 0.000985
Dowagiac	16	2	0.1250 ± 0.1064	0.125000 ± 0.202014	0.000119 ± 0.000216

*Note*: *N* and *N*_h_ refer to the number of individuals and number of haplotypes per sample, respectively. Standard deviations are provided for each estimate.

AMOVA was used to partition genetic variance into within and among sample components, identifying significant differences among samples (*F*_ST_ = 0.457, *p* < .0001, 54.3% of the variation). Further subdivision to assess levels of variation within and among two sets of drainages (Lake Michigan vs. Lakes Huron, St Clair, and Erie) indicated that variation was largely attributable to differences among samples within those two drainage groups (*F*_SC_ = 0.435, *p* < .0001, 41.9% of the variation), not differences among them (*F*_CT_ = 0.038, *p* = .19, 3.8% of the variation). The lack of differentiation among the drainage groups appears to be driven by a shared haplotype (U) between Stony Creek and the Kalamazoo River. If Stony Creek is removed from the analysis, differences among drainage groups becomes significant (*F*_SC_ = 0.565, *p* < .0001, *F*_CT_ = 0.100, *p* < .023). However, differences among samples within the groups and within populations explain a much larger proportion of the variation (51.0% and 39.2%, respectively) than differences among groups (9.9%).

**FIGURE 8:**
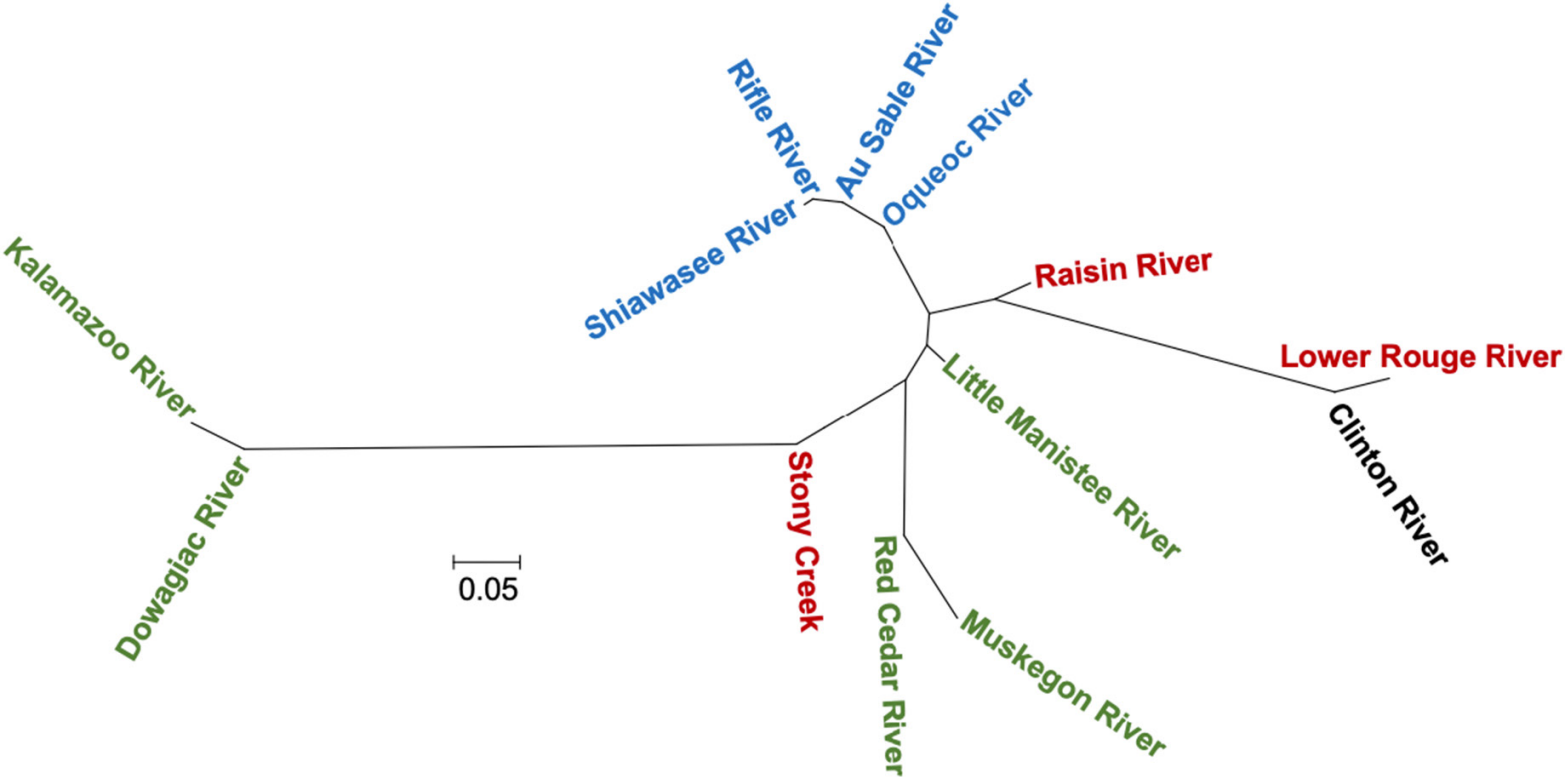
Neighbor‐joining tree based on pairwise *F*_ST_ estimates for *E. nigrum*. Label color indicates major basin: Black – Lake St. Clair, Green – Lake Michigan, Red – Lake Erie, Blue – Lake Huron.

Pairwise estimates of *F*_ST_ (Table 6) were used to construct a neighbor‐joining tree for *E. nigrum* population samples, revealing similarities among the locations within the major basins (Figure 8). Samples from Lake Huron River drainages were like each other while those of the other basins exhibited more divergence among populations. Samples from the Muskegon River population, a Lake Michigan drainage, and the Raisin River, a Lake Erie drainage, clustered with samples from the Lake Huron basin instead of more geographically proximate samples because of the high frequency of Haplotype A (Table 4; Figure 7). Drainages of Lake St. Clair and Lake Erie were generally intermediate to those from Lakes Huron and Michigan; however, samples from these basins and Lake Michigan have long terminal branches reflecting the high frequency of private alleles at these locations.

**TABLE 6:** Pairwise estimates of *F*_ST_ for *E. nigrum* populations.

	RN	LR	CL	SH	RF	AS	OQ	SC	LM	MU	RC	KZ
LR	0.59300											
CL	0.42047	0.62722										
SH	0.06667	0.57570	0.41789									
RF	0.04725	0.56864	0.43515	0.01816								
AS	0.02778	0.58206	0.41189	0.04210	0.02262							
OQ	0.02268	0.46580	0.35012	0.03354	0.03996	0.00709						
SC	0.15878	0.28477	0.28823	0.16683	0.22339	0.15921	0.10720					
LM	0.41412	0.55534	0.53071	0.42083	0.49298	0.41102	0.25814	0.11338				
MU	0.12001	0.50833	0.39239	0.12551	0.13964	0.10536	0.07027	0.15679	0.38089			
RC	0.76347	0.83729	0.69670	0.73357	0.71050	0.75303	0.59165	0.27691	0.55047	0.58243		
KZ	0.76165	0.81794	0.77040	0.75823	0.78376	0.75884	0.67100	0.19774	0.41001	0.71184	0.83311	
DG	0.77181	0.84190	0.70357	0.74241	0.71805	0.76167	0.60429	0.28644	0.56226	0.63595	0.93103	0.8381

*Note*: Significant values are underlined. See Section 2 for locality abbreviations.

**FIGURE 9:**
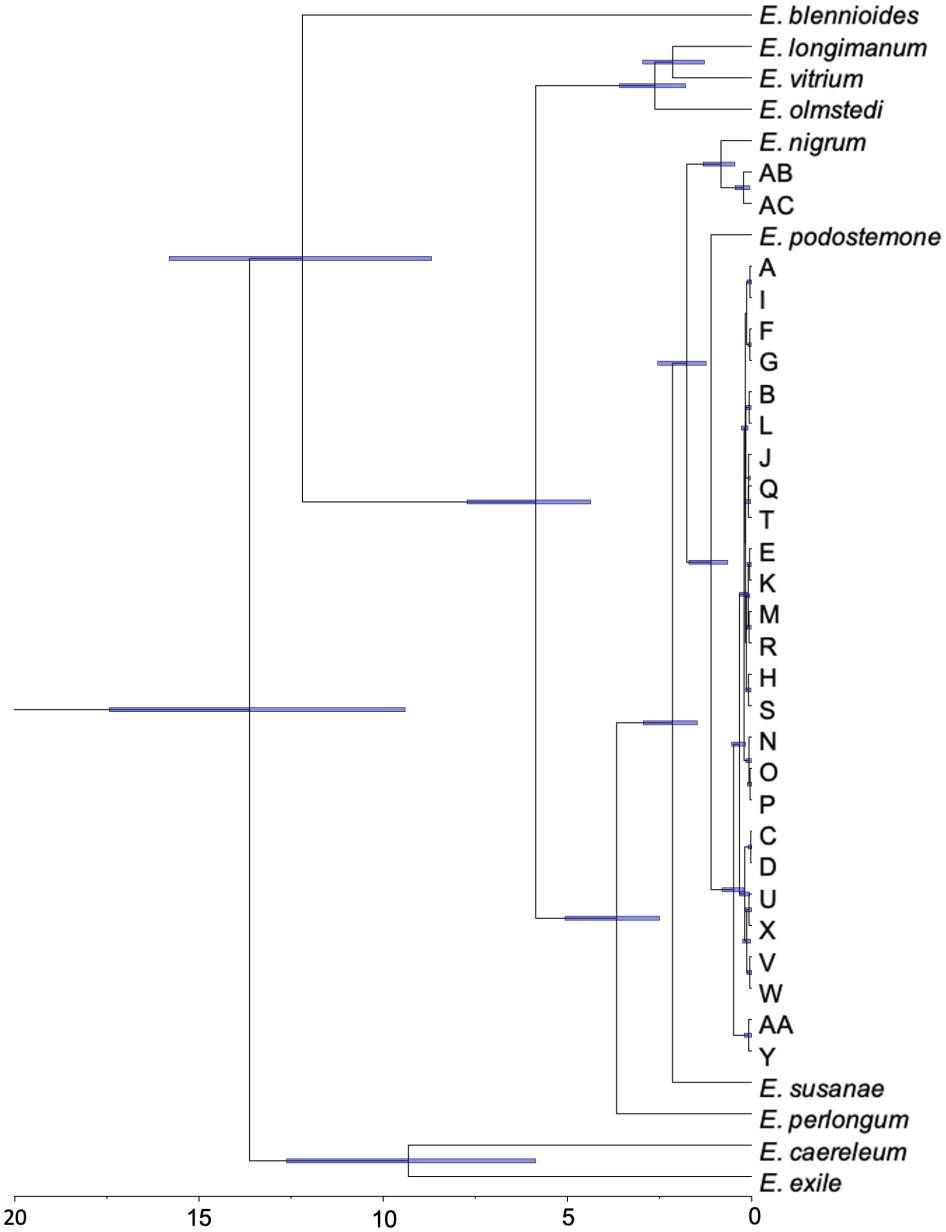
Time tree generated by molecular divergence analysis with *BEAST for *E. nigrum* haplotypes including related *Etheostoma* species. Blue bars represent the 95% HPD Scale is shown in millions of years before present.

Estimates of molecular divergence were calculated using *BEAST to better understand the context of highly divergent *E. nigrum* haplotypes. The resulting tree generated with TreeAnnotator and visualized with FigTree is shown in Figure 9. *Etheostoma nigrum* haplotypes from these populations show two major mtDNA lineages (Haplotypes A – AA and AB – AC) which diverged approximately 1.8 mya (95% HPD 1.2–2.6 Ma). There are two groups in the main lineage (A – X, Y – AA) which diverged approximately 500,000 years ago (95% HPD 0.25–0.83 Ma). Within this main lineage, divergence between haplotypes range from 70,000 to almost 200,000 years ago. The sister group contains haplotypes Y and AA which are about 90,000 years diverged. The divergent lineage that contains haplotypes AB and AC shares its most recent common ancestor with the *E. nigrum* individual from the Embarras river, with estimated divergence time of approximately 600,000 years ago.

## 4 DISCUSSION

Patterns of genetic variation in *N. melanostomus* and *E. nigrum* were explored using a variable mitochondrial DNA gene to assess the levels of divergence within and among populations. It was predicted that, as a recently introduced species, *N. melanostomus* would show limited divergence among populations and low genetic diversity. As a native species with more limited dispersal, greater diversity within and more divergence among *E. nigrum* populations was predicted. The results were consistent with these expectations. It was also predicted that *E. nigrum* populations of east and west basins of the Lower Michigan would be significantly different due to distinct colonization sources, but those from eastern and western lake basins were not significantly different. Despite this result, the distribution of genetic diversity provided insight into the geologic and ecological factors that may have shaped the population structure in *E. nigrum*.

### 4.1 Neogobius melanostomus

ND2 sequences from *N. melanostomus* showed substantially less diversity than *E. nigrum*. Invasion of the Great Lakes by *N. melanostomus* is recent, arriving in ballast water from the Black Sea circa 1990. Previous work has found that, as it expanded its range within the Great Lakes, *N. melanostomus* has retained levels of genetic variation on par with source populations (Brown & Stepien, 2009). Population genetic studies of *N. melanostomus* in more recently established stream populations show that *N. melanostomus* is expanding its range without founder effects (Bronnenhuber et al., 2011). Bronnenhuber et al. (2011) also found *N. melanostomus* populations lacked genetic structure. Consistent with this result, there were few significant differences among populations of *N. melanostomus* and no significant geographic structure. Brown and Stepien (2008) found limited structure among *N. melanostomus* populations in the Great Lakes, with this result primarily driven by differences between populations (Lake Ontario and Saginaw Bay) which were not included in this study. Additional statistically significant structure in these previous studies may also have been driven by low‐frequency private alleles in lake populations which may have been missed here due to the small number of samples and their sizes, or that these rare alleles may not yet be present in these more recently established stream populations. The small number of ND2 haplotypes and dominance of a single haplotype identified in *N. melanostomus* is consistent with other previous work examining levels of mtDNA variation in *N. melanostomus* (Brown & Stepien, 2008; Stepien & Tumeo, 2006).

*Neogobius melanostomus* has dispersed rapidly throughout the Great Lakes (Kornis, Mercado‐Silva, & VanderZanden, 2012), resulting in high levels of gene flow, and the homogeneity and low diversity of samples around the Great Lakes is consistent with expectations. Established populations of *N. melanostomus* are less prone to dispersal and favor residents (Thorlacius et al., 2015); however, even very low levels of migration can provide sufficient gene flow to prevent population divergence at presumably neutral loci like these (Newman & Tallmon, 2001; Slarkin, 1985). As a high dispersing species not restricted by habitat, migration will likely be a persistent, homogenizing force in *N. melanostomus*. This situation has been aided by human activity as secondary spread of *N. melanostomus* to Michigan's inland waterways has occurred due to the accidental movement of *N. melanostomus* in live bait used by anglers (Sard et al., 2019). Populations found where barriers such as dams prevent natural migration will likely be distinct from connected populations due to founder effects and genetic drift. However, where migration and human activity are a constant source of gene flow, the lack of structure observed here will likely persist.

### 4.2 Etheostoma nigrum

As a native species, the distribution of *E. nigrum* has been shaped, in part, by the action of glaciers. Ancestors of this species likely colonized the early Great Lakes region more than 10,000 years ago as glaciers receded, and the modern lake basins were formed (Bailey & Smith, 1981). In addition, *E. nigrum* tends to be habitat restricted, occupying shallow, slow‐moving stretches of streams and rivers. Such preferences have been shown to make fishes less likely to move among rivers through downstream dispersal and more likely to diverge (Tibbets & Dowling, 1996). *Etheostoma nigrum* also have relatively low fecundity with males guarding nests, characteristics which may limit gene flow (Stepien et al., 2007; Turner & Trexler, 1998). This is reflected in results of the AMOVA, where significant differences were mainly driven by variation among individual river drainages within lakes basins, but not between east and west basins as expected, even if the population with the divergent haplotypes (Stony Creek) is removed from the analysis. Westbrook (2012) identified a similar pattern in Wisconsin drainages of the Great Lakes, in which geographic isolation resulted in limited gene flow and significant divergence among *E. nigrum* populations in different drainages.

For species limited in their ability to disperse by suitable habitat, isolation of individual populations can lead to divergence. Dowling et al. (2015) highlighted the importance of local forces in shaping population structure among populations of three species of roundtail chub (*Gila intermedia*, *G. nigra*, and *G. robusta*), noting high levels of population level divergence that could obscure broader historical factors. It is possible that the east and west basins of the Great Lakes were colonized by distinct populations of *E. nigrum*, but over time evolutionary processes may have driven divergence among local populations such that differences among broader hierarchical categories cannot be detected (Hedrick, 1999). Similar patterns have been observed for other species including smallmouth bass (*Micropterus dolomieu*, Stepien et al., 2017), white sucker (*Catostomus commersoni*, Lafontaine & Dodson, 1997), and mottled sculpin (*Cottus bairdii*, Homola et al., 2016).

Despite isolation due to limited dispersal, more recent shifts in hydrology and geologic features in the region may provide paths of dispersal that facilitate gene flow. These shifts may explain some of the patterns observed in *E. nigrum*. Ray et al. (2006) examined mtDNA variation among rainbow darter (*Etheostoma caeruleum*) populations (an ecologically similar species to *E. nigrum*) and found that sampled Wabash River populations grouped with samples from both Lakes Michigan and Erie. Stream capture of parts of the Wabash River (a tributary of the Ohio River) by the Maumee River (Figure 1) may have facilitated gene flow between the two basins, as evidenced by similarity of haplotypes with Stony Creek (Lake Erie drainage) and the Wabash River drainage (Figure 6). *Etheostoma nigrum* populations appear to have followed a similar colonization pattern in which colonization of Lake Erie occurred via the captured portion of the Wabash River and propagated throughout the Lake Erie watershed, reducing structure based on east/west basins. The molecular dating analysis placed the divergence of this Wabash River‐derived lineage during the Pleistocene, ~1.8 mya.

Construction of a neighbor‐joining tree using *E. nigrum* population samples also revealed similarities among samples within major basins, especially Lake Huron; however, one Lake Michigan drainage (Muskegon River) grouped with Lake Huron drainages (Figure 8). This grouping appears to be the result of the higher frequency of the “A” haplotype in the Muskegon River, which is rare in the other drainages of the western basin and common in the eastern basin drainages. Given the close proximity of the headwaters of the Muskegon River to the headwaters of several Lake Huron drainages (Figure 1) and the low, marshy topography of the region, it is possible that connections historically existed and allowed gene flow between basins in this region. During high water events, connections may temporarily exist in the present day.

Despite their recent origin, there is a high level of ND2 diversity within and among these *E. nigrum* populations (*F*_ST_ = 0.406, *p* < .0001 and mean pairwise differences ranging from 0.125–9.66). Based on *BEAST analysis, emergence of the most recent haplotypes predates formation of the modern Great Lakes (70,000 to 200,000 years ago), indicating that some haplotypes may have been found in different refugia. Additionally, two highly divergent haplotypes grouped closely with geographically distant populations. This would suggest that much of this diversity was introduced from populations maintained along the glacial front, rather than evolving in situ. Given that only one *E. nigrum* ND2 haplotype was available in Genbank and already included in the analysis, further exploration of source populations using ND2 was not available at this time. However, many cyt*b* sequences are available and a sample of these from around the Midwest (MacGuigan et al., 2023), along with a subset of cyt*b* sequences from the Rouge and Clinton River individuals were used in a maximum‐likelihood tree which yields a large clade with minimal structure. Individuals from these Rouge and Clinton haplotypes group with geographically diverse locations including the Ohio River, Wabash River, Kentucky River, and Wisconsin River, including a shared haplotype between the Rouge, Ohio, Wabash, and Embarrass Rivers (data not shown, available upon request). The minimal structure in cyt*b* haplotypes supports the conclusion from the ND2 analysis that diversity was contributed by multiple source populations along the glacial front.

Additional unexpected patterns were noted in the maximum‐likelihood tree of *E. nigrum* haplotypes and related taxa (Figure 6). The clustering of *E. podostemone* within *E. nigrum* haplotypes is inconsistent with current darter phylogenies. Darters are a highly speciose group and their taxonomy is not fully resolved (Near et al., 2011). MacGuigan and Near (2018) characterized the group which includes *E. nigrum* using next generation sequencing data, yielding two major lineages, one containing *E. nigrum*, *E. olmstedi*, and *E. perlongum*, and the other *E. podostemone* and *E. longimanum* (Figure 6b). The results from this analysis (Figure 6a) are not generally consistent with this result as the two lineages identified here include (1) *E. nigrum*, *E. perlongum*, and *E. podostemone* and (2) *E. olmstedi* and *E. longimanum*. Differences between these studies may reflect differences mitochondrial inheritance patterns and/or introgression among species compared to nuclear genes. Understanding reasons behind the discordance between mtDNA and nuclear genes requires further study.

The position of *E. podostemone* is especially interesting as it is found clustered within a group of *E. nigrum* haplotypes. *Etheostoma podostemone* is localized to a small and isolated region in Virginia and North Carolina, distant from the localities sampled here. Heckman et al. (2009), using nuclear genes and the mitochondrial cyt*b* gene, found a similar pattern of nesting of *E. podostemone* among *E. nigrum* haplotypes from the Mississippi River, Mobile River, and the Great Lakes. MacGuigan et al. (2023) also identified the nesting of *E. podostemone* within *E. nigrum* with mtDNA, while the phylogeny generated from restriction site associated DNA sequencing (RADseq) grouped *E. podostemone* most closely with *E. longimanum*, forming a sister clade to *E. nigrum*. This pattern may reflect ancient mitochondrial introgression, a process that has often been identified as an important mechanism in the evolution of *Etheostoma* species (Bossu & Near, 2009; MacGuigan & Near, 2018; Ray et al., 2006).

## 5 CONCLUSIONS

It was predicted that, as a recently introduced species, *N. melanostomus* populations would show low diversity and limited geographic structure. The results were consistent with this hypothesis. *Neogobius melanostomus* populations were dominated by a single mtDNA haplotype and few statistically significant differences among locations as detected using F‐statistic analysis. This result reflects the history of round goby in the Great Lakes, having been recently founded from a single source population, followed by rapid dispersal through the region.

This is the first study to examine the population genetics of *E. nigrum* in Lower Michigan, with population genetic structure of *E. nigrum* in Lower Michigan offering insight into the historic processes that shaped the geographic distribution of the species. Native *E. nigrum* is more likely to exhibit geographic differences among and higher levels of genetic diversity within populations than *N. melanostomus*, reflecting its past geological and evolutionary history. This study identified differences among populations within basins as a significant source of variation with limited divergence among regions. Contrary to predictions based on the findings of previous studies (Dowling et al., 1997; Gach, 1996; Stepien et al., 2009), there were not significant differences between the eastern and western basins of the Lower Michigan; however, multiple mtDNA lineages found within *E. nigrum* populations suggests that multiple source populations colonized the region as the glaciers receded, and historic routes of gene exchange among basins may have reduced the level of genetic differences between them. Given this pattern was consistent with that of an ecologically similar species (*E. caeruleum*), it may follow for other similar species as well. As this study only included streams in Lower Michigan, future research could expand to a broader geographical range to include more of the Great Lakes drainage and as and additional markers, allowing for greater historical perspective. This also informs future work comparing gene expression differences in *E. nigrum* and *N. melanostomus* populations, allowing evolutionary history to be considered as a source of variation in the patterns of gene expression within and between these species.

## AUTHOR CONTRIBUTIONS

**A. J. Wicks:** Conceptualization (equal); data curation (lead); formal analysis (lead); investigation (lead); methodology (equal); writing – original draft (lead); writing – review and editing (lead). **M. Bowman:** Data curation (supporting); investigation (supporting); writing – original draft (supporting); writing – review and editing (supporting). **T. E. Dowling:** Conceptualization (equal); formal analysis (supporting); supervision (lead); writing – original draft (supporting); writing – review and editing (supporting).

## CONFLICT OF INTEREST STATEMENT

The authors declare no competing interest.

## Data Availability

All ND2 haplotype sequences for *E. nigrum* and *N. melanostomus* have been submitted to GenBank under accession numbers OR777617–OR777644 and OR795530–OR795534, respectively.
